# Advantages and Challenges of Using ctDNA NGS to Assess the Presence of Minimal Residual Disease (MRD) in Solid Tumors

**DOI:** 10.3390/cancers13225698

**Published:** 2021-11-14

**Authors:** Lionel Larribère, Uwe M. Martens

**Affiliations:** 1 Department of Hematology and Oncology, Cancer Center Heilbronn-Franken, SLK Clinics Heilbronn GmbH, 74078 Heilbronn, Germany; uwe.martens@slk-kliniken.de; 2 Skin Cancer Unit, German Cancer Research Center (DKFZ), 69120 Heidelberg, Germany; 3 Department of Dermatology, Venereology and Allergology, University Medical Center Mannheim, Ruprecht-Karl University of Heidelberg, 68167 Mannheim, Germany; 4 MOLIT Institute for Personalized Medicine GmbH, 74076 Heilbronn, Germany

**Keywords:** liquid biopsy, ctDNA, minimal residual disease, adjuvant therapy

## Abstract

**Simple Summary:**

Minimal residual disease (MRD) represents a status of the disease which is assumed to still be present in the body until it is clinically observed by radiology. At this time point, the tumor relapse is present and a new clinical decision should be taken. However, there is currently no official biomarker which can efficiently predict a relapse after a curative-intent surgery or treatment. This unmet clinical need would benefit from such a biomarker as it would help guiding the decision on adjuvant therapy. The possibility to use the liquid biopsy technology in order to measure non-invasive circulating tumor DNA (ctDNA) in the patient’s blood opens a new avenue to the establishment of this biomarker. In this review we summarize the current knowledge on ctDNA detection by NGS as a tool to assess the presence of MRD as well as the clinical trials focusing on the clinical utility of the method.

**Abstract:**

The ability to detect minimal residual disease (MRD) after a curative-intent surgery or treatment is of paramount importance, because it offers the possibility to help guide the clinical decisions related adjuvant therapy. Thus, the earlier MRD is detected, the earlier potentially beneficial treatment can be proposed to patients who might need it. Liquid biopsies, and in particular the next-generation sequencing of circulating tumor DNA (ctDNA) in the blood, have been the focus of an increasing amount of research in the past years. The ctDNA detection at advanced cancer stages is practicable for several solid tumors, and complements molecular information on acquired therapy resistance. In the context of MRD, it is by definition more challenging to detect ctDNA, but it is technically achievable and provides information on treatment response and probability of relapse significantly earlier than standard imaging methods. The clinical benefit of implementing this new technique in the routine is being tested in interventional clinical trials at the moment. We propose here an update of the current use of ctDNA detection by NGS as a tool to assess the presence of MRD and improve adjuvant treatment of solid tumors. We also discuss the main limitations and medium-term perspectives of this process in the clinic.

## 1. Introduction

The field of liquid biopsy is evolving at a fast pace, and the multitude of analytes present in the different bodily fluids (especially in the blood) favors the development of a large panel of detection methods. For example, cell-free DNA (cfDNA) circulates in the peripheral blood of all individuals, due to its release from apoptotic or necrotic cells [1]. In the situation of cancer, tumor cells will release so-called circulating tumor DNA (ctDNA) in the blood, representing a rather minor fraction of cfDNA [2]. Additional mechanisms such as a defect in the circulating DNA clearance by macrophages may account for the observed higher ctDNA levels in cancer patients [3]. The detection of ctDNA at advanced cancer stages has greatly improved in recent years, and its clinical utility has been demonstrated in some cases. Indeed, the retrospective CORRECT trial could predict the clinical activity of regorafenib and assess the prognosis in patients with metastatic colorectal cancer [4]. Resistant EGFR mutations were identified in patients with metastatic colorectal cancer under anti-EGFR treatment [5]. ctDNA genotyping also significantly shortened the screening duration and improved the trial enrollment rate without compromising treatment efficacy compared to tissue genotyping in a patient cohort (n = 1687) with advanced gastrointestinal cancers [6]. However, at early stages of the disease or after a curative-intent surgery or treatment, the detection of extremely low levels of ctDNA present in a blood sample is challenging due to its stochastic distribution and will depend not only on the assay’s sensitivity but also on the preanalytical sample preparation. Moreover, the assessment of minimal residual disease (MRD) at the time of the ctDNA measurement can only be assumed until it becomes clear that a relapse is clinically diagnosed.

**Figure 1:**
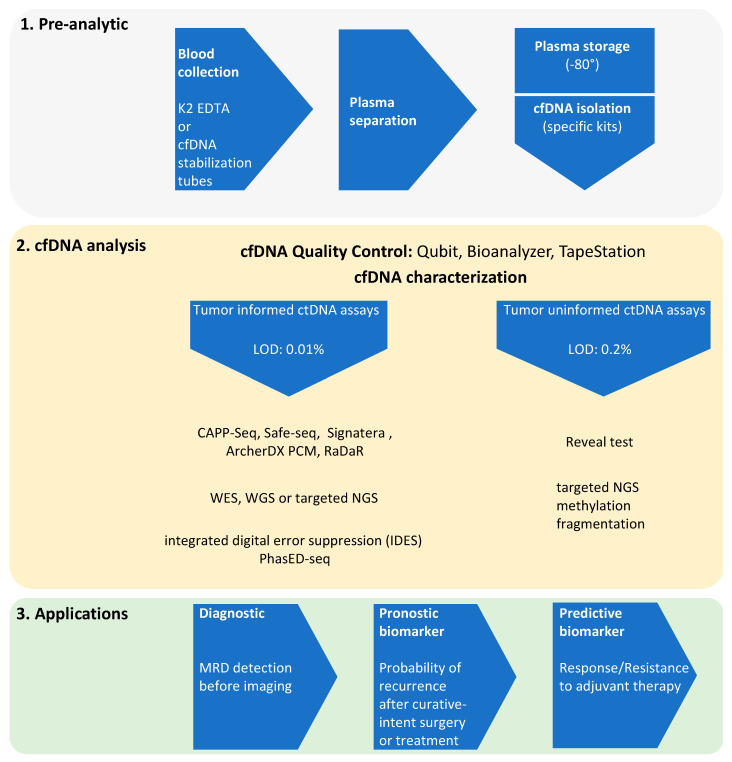
Workflow of ctDNA NGS in the settings of minimal residual disease (MRD).

Historically, the first FDA approval for the use of MRD in treatment decisions was decided in 2018 in the context of B-cell precursor acute lymphoblastic leukemia. Since then, two classes of ctDNA-based assays have been developed and applied to solid tumors: (1) Tumor-informed assays such as CAPP-Seq, Safe-seq, Signatera or ArcherDX. Usually designed for the surveillance in patients whose mutations are known from the tumor, these assays have a limit of detection as low as 0.01% variant allele frequency (VAF) [7,8,9,10,11,12]. Higher sensitivities have been reported with the CAPP-seq technique by the implementation of the integrated digital error suppression (IDES) barcoding strategy, or more recently of a process called PhasED-seq based on the identification of several somatic variants on the same DNA fragment [13,14]. The CAPP-seq technique is described as an ultrasensitive and cost-efficient method for the quantification of ctDNA (Figure 1). (2) Tumor-naïve assays: these broad panel-based sequencing assays integrate both genomic alterations and methylation status, are used for genotyping or early detection and reach a detection limit of about 0.2% (Guardant Health Reveal test) [15]. A nonexhaustive list of the different assays used to detect ctDNA as evidence for the presence of MRD is shown in Table 1.

Due to the wide range of sensitivity or positive predictive value (PPV) which have been reported for all these assays, systematically comparing the results is delicate. These data point out the need for complex mathematical algorithms to reach confident results from panel sequencing in recurrence monitoring [16] and demonstrate the lack of an agreed gold standard for minimal residual disease. As such, the detection of tumor-informed ctDNA biomarkers in solid tumors compared to haematological disorders is still lacking irrefutable evidence to represent MRD and should be used with caution before clinical decision.

In this review, we summarize the current updates on ctDNA sequencing assays used as a tool to detect the potential presence of MRD and to monitor the response to adjuvant therapy. We also discuss the main limitations of the technique and its future horizon. For better clarity, we chose to focus on non-small-cell lung cancer (NSCLC) and colorectal cancer (CRC), however ctDNA detection after definitive treatment was strongly associated with distant recurrence also in esophageal or breast cancer [17,18,19,20].

## 2. ctDNA NGS and MRD Evidence

Minimal residual disease (MRD) corresponds to a small number of otherwise undetectable cancer cells that still remain in the patient after curative-intent surgery or treatment. The possibility to detect ctDNA after definitive treatment and to robustly associate with future radiological relapse could improve the choice of the next treatment line. Although the probability of severe adverse effects of current therapies is lower than in the past, most treatments are still based on strict chemotherapy protocols. It is thus important to avoid unnecessary adjuvant chemotherapy whenever a patient who will likely not benefit, can be identified. Therefore, the two main advantages of correlating early ctDNA detection to future relapse are 1) to identify better patients who will actually benefit from adjuvant therapy, and 2) to use the high specificity of the ctDNA test compared to the current assessment with clinical risk factors.

### 2.1. Evidence of MRD in NSCLC

ctDNA is strongly prognostic in patients with localized NSCLC, and in this situation, ctDNA analysis provides additional information for clinical decisions on consolidation therapy after surgery. The TracerX consortium was one of the first studies to set up a ctDNA detection method during surveillance of patients with early stage NSCLC, which preceeds imaging-based recurrence [10]. Because of an insufficient limit of detection (LOD), investigators decided to use prior knowledge of tumor-tissue mutations to increase the sensitivity of the CAPP-seq test (this point is discussed in paragraph 3) (Figure 1). The main result of TracerX was that almost all patients with postoperative relapses had detectable ctDNA before or at the time of recurrence diagnosis at follow-up. These data suggested that the early detection of ctDNA was associated with aggressive tumor biology and faster tumor growth [21]. Another result of the study was to highlight that preoperative ctDNA dynamics influence MRD lead times. However, the clinical significance of such molecular information is still a matter of investigation, and the aim of current trials within the TracerX consortium is to test whether postoperative chemotherapy is specifically improving the outcome of patients with detected ctDNA. In a similar study, Chauduri et al. aimed at identifying post-treatment MRD in NSCLC patients earlier than the current SOC, which would allow a time window for treatment, during which tumor burden and heterogeneity are still low. They concluded that both node-positive and node-negative patients with stage I–III NSCLC may benefit from personalized adjuvant therapy. In particular, when assessed for actionable mutations and mutational load in ctDNA, patients for whom tissue material was not available could benefit from early tyrosine kinase inhibitors (TKI) or immune checkpoint inhibitors (ICI) [22]. The main differences with the TracerX study were 1) a prognostic value analysis of the ctDNA detection, assumed as MRD was performed, and 2) patient cohorts with different stages were involved. Recently, a study on 84 patients with localized NSCLC showed a ctDNA detection rate ranging from 42% to 88% (stage I to III, respectively). Interestingly, this rate varied according to histology, with 43% of adenocarcinoma versus 95% of nonadenocarcinoma samples [23]. In summary, these data show that measurement of ctDNA, the sensitivity of which increases with the number of targets, is able to assess the potential presence of MRD which will allow clinicians to identify which patients are likely to relapse in the future. A list of current clinical trials testing the utility of ctDNA detection in NSCLC in the adjuvant setting is presented in Table 2.

### 2.2. Evidence of MRD in CRC

Surgery of CRC at stages I and II is often curative. At higher stages, adjuvant chemotherapy can reduce the risk of recurrence [24]. Nevertheless, no effective biomarker is available to predict the risk of recurrence in MRD-positive patients. At stage II and III, ctDNA positivity after adjuvant treatment completion is associated with poorer relapse-free survival (RFS) [9,25,26]. These data suggest that persistent detection of ctDNA post-treatment reflects the presence of micrometastatic disease, which ultimately is the source of clinical recurrence. Most active drugs in the advanced setting did not bring clinical benefit in the adjuvant trials [27,28,29]. The difficulty of designing clinical trials in the adjuvant settings lies in the fact that they require large sample sizes and long periods of follow-up (in order to reach progression free survival (PFS) or overall survival (OS) endpoints), because patients who are at risk of recurrence cannot be clearly identified. New trial design based on the high positive predictive value of ctDNA (almost all patients with positive ctDNA assay eventually develop clinical recurrences) is probably necessary in order to evaluate novel agents in these patients with the highest risk of recurrence, while sparing toxicities in those who are not at such a high risk. Several trials are currently investigating the benefit of additional therapy to reduce the risk of relapse in CRC based on ctDNA evaluation (Table 1). The TRACC study (NCT04050345), for example, designed on stage II-III CRC, patients had a median follow up of survival of about 15 months. In total, 6 out of 14 (43%) MRD-positive patients relapsed whereas only 8 out of 93 (9%) MRD-negative patients did (HR: 10; 95% CI: 3.3–30; *p* < 0.001). ctDNA status was found to be the most significant prognostic factor associated with RFS (HR: 28.8, 95% CI: 3.5–234.1; *p* < 0.001) and the authors concluded that post-operative ctDNA analysis with a tumor-informed assay enables detection of CRC patients at high risk of recurrence [30].

Tumor-specific DNA methylation is described during early tumorigenesis, and presents high potential for early detection but also for tumor localization [31]. The analysis of epigenetic features of cfDNA or its fragmentation pattern leads to increased sensitivity of the ctDNA detection tests [32] and has led to the development of plasma-only assays to assess MRD. Indeed, samples from 103 patients with colorectal cancer undergoing curative-intent surgery were analyzed with plasma-only ctDNA assay integrating genomic and epigenomic cancer signatures (Reveal test, Guardant Health) to enable a tumor-uninformed analysis reflecting the presence of MRD. This new design increased the sensitivity by 25%–36% compared to the analysis of genomic alterations alone. The authors showed that out of 84 patients, after completion of therapy (surgery or adjuvant), 14 had detectable ctDNA of which all had recurrences, whereas 49 had no ctDNA of which 12 relapsed. This data demonstrated favorable sensitivity for recurrence detection with surveillance samples and favorable specificity for recurrence detection following completion of definitive therapy in colorectal cancer patients, comparable with tumor-informed approaches [15]. Interestingly, a similar study performed a tumor-informed approach to detect ctDNA in CRC patients and reached the conclusion that postoperative ctDNA status can identify patients with a high risk of recurrence, and that longitudinal monitoring using ctDNA can guide treatment decisions [33].

Two interventional trials have been derived from the LUNAR program of Guardant Health. COBRA (NCT04068103) is an escalation trial designed to direct adjuvant chemotherapy to stage II colon cancer patients with ctDNA-based MRD positivity, compared to active surveillance of MRD-negative patients. PEGASUS (NCT04259944) however, is a de-escalation trial designed to switch chemotherapy regimens based on MRD status every 3 months. Escalation and de-escalation adjuvant chemotherapies in stage II and III CRC patients are also being evaluated in the randomized DYNAMIC trials (DYNAMIC-II: actrn12615000381583; DYNAMIC-III: ACTRN12617001566325) based on MRD status. In the ctDNA-informed arm, MRD-positive patients will receive standard 5FU-based chemotherapy while MRD-negative patients will receive no adjuvant. The other arm will receive standard chemotherapy and will be regularly monitored for ctDNA presence. 

### 2.3. MRD Evidence to Guide Immunotherapy

In order to better predict the patient’s response to the recently successful immune checkpoint blockade, a few pan-cancer biomarkers have been proposed [34]. One example is the Microsatellite Instability (MSI) status, based on higher prevalence of mutations in microsatellites, which is the consequence of DNA mismatch repair deficiency and which seems essential for antitumor immunity [35,36]. The other example is the tumor mutational burden (TMB) which has shown predictive value for ICI in some NSCLC studies [37]. The detection of both MSI and TMB biomarkers is being currently investigated in blood [38,39,40,41,42].

The randomized clinical trials POPLAR (NCT01903993) and OAK (NCT02008227) have shown significant improvement in survival after ICI (atezolizumab) therapy in previously treated NSCLC patients. A retrospective analysis of these two trials showed an association between ctDNA-based TMB and favorable clinical outcome after second-line ICI treatment compared to chemotherapy (TMB > 16 mut/Mb; median OS 13.5 versus 6.8 months, respectively) [43]. Nevertheless, the utility of blood TMB as a predictive biomarker needs to be confirmed in prospective clinical trials. A report at the ESMO 2021 conference presented the feasibility of a tumor-agnostic ctDNA analysis to test the response to ICI in the adjuvant setting (NCT03832569). Somatic tissue mutations were assessed using MSK-IMPACT, and ctDNA was assessed using FoundationOne, Guardant360 or MSK-ACCESS. Investigators concluded that ctDNA detection predicted the risk of relapse in resected MSI-high patients, and plan now to assess the response of these MRD positive patients to ICI [44]. An example of a trial assessing adjuvant immunotherapy in NSCLC patients is the phase-3 PACIFIC trial (NCT02125461). The study tested the impact of PD-L1 inhibitor (durvalumab) in stage III patients without progression after chemoradiotherapy (CRT), and showed a reduced median PFS by almost 3 times in the durvalumab-treated group. These results led to the FDA approval of durvalumab after chemoradiation for unresectable stage III NSCLC [45,46]. Nevertheless, it should be noted that overall, the largest group of patients did not derive benefit from consolidation ICI. Moreover, more than 30% of patients receiving durvalumab experienced at least one grade 3 or 4 adverse events.

In order to better identify responders, Diehn and colleagues decided to assess ctDNA to predict patients’ response to adjuvant ICI (in this case, following CRT). They performed a retrospective study of 62 patients with stage III NSCLC (37 who received CRT without further treatment and 25 who received CRT followed by consolidation with an ICI) [47]. The first result showed that ctDNA negative patients did not benefit from ICI consolidation compared to those with no further treatment, suggesting that ctDNA testing would help spare this therapy to patients who are unlikely to respond. The second result was to identify these patients with positive ctDNA after CRT whose ctDNA became undetectable after ICI treatment and who showed clinical benefit. In this study, the median collection time for early treatment with ICI was 11 weeks, but the authors suggested that respondent patients could be identified as early as 8 weeks based on other studies [48,49,50]. Although TMB has been associated with response to ICI in some NSCLC studies, this biomarker was not associated with the consolidation setting of this study. The ongoing randomized MERMAID trials (NCT04385368; NCT04642469) are assessing the impact of durvalumab and chemotherapy in the context of adjuvant therapy in stage II-III MRD-positive NSCLC patients compared to SOC adjuvant chemotherapy. The primary endpoint will be PFS in patients with PD-L1 positivity. In the same line, a recent Canadian study is measuring ctDNA during surveillance of NSCLC patients who are given different chemotherapies or ICI as adjuvant therapy (NCT04966663). Of note, ctDNA detection was also reported in patients with long-term response to ICI (more than one year) [51].

Recently, a new approach to assess MRD and predict response to ICI with ctDNA was proposed and included additional analysis of circulating immune cells [49,52,53]. Based on pre- and early-treatment blood samples and on a new statistical model, authors could include these parameters in a single biomarker capable of robustly identifying which NSCLC patients will achieve durable clinical benefit (DCB) from ICI. They suggested that this new biomarker has a high prognostic value and propose to use it in complement to radiologic surveillance in NSCLC patients treated with PD1 or PD-L1 blockade [52]. In the same line, melanoma patients responding to ICI have shown increased T-cell receptor sequences, an expansion of a subset of cytotoxic memory effector T cells, or increased large T-cell clones in their blood [54,55]. Thus, concurrent ctDNA and T-cell expansion analysis appears to be very important for future prediction of response to ICI. More efforts are still needed to be able to predict the response early enough, as a fraction of MRD-negative patients relapse regardless. For instance, performing sequential ctDNA testing after chemoradiotherapy could be an option to stratify patients to postadjuvant therapies [56].

## 3. Challenges of the ctDNA-Based NGS Technology

Extensive work has been performed to compare the concordance between tumor and plasma-derived NGS analyses. Indeed, there are technical and biological factors which account for the generation of false-negative and false-positive results in ctDNA analyses.

### 3.1. False-Negativity Rate

The first technical challenge is the total volume of plasma derived from a classical 10 mL blood draw. In the plasma volume of one sample, the total number of genome copies is very limited, and therefore so is the number of a particular variant of interest. Moreover, the amount of a clonal variant in the sample will be different than that of a subclonal variant. The probability to catch that particular variant in the sample comes down to a statistical distribution, and might be demanding when the VAF lowers close to the LOD. In addition, the tumor fraction of cfDNA varies between cancer entities or even between patients with the same entity.

Some false-negative results simply cannot be prevented due to biological factors such as low DNA shedding of certain tumors or the location of metastases itself. Indeed, it has been described that metastatic CRC patients with liver metastases have higher chance to carry ctDNA in the blood than those with nodal or lung metastases [57,58,59,60]. Interestingly, studies based on ctDNA have also shown higher sensitivity for distant compared to locoregional metastases detection [17,61]. It has been postulated that micrometastases represent a higher tumor burden than residual local disease and can therefore shed higher ctDNA levels.

Several technical improvements for the different ultradeep sequencing methods used to analyze ctDNA are being developed and require complex algorithms [9,62]. It appears that the size selection of cfDNA fragments will play a bigger role in the characterization of the isolated cfDNA.

### 3.2. False-Positivity Rate

The difficulty to identify true biological variants is mainly due to the risk of introducing errors during library preparation or the sequencing process itself, and requires the additional steps of multiple mutation-enrichment methods and error-suppression strategies such as the introduction of molecular barcodes or bioinformatic analysis pipelines [21,63,64] (Figure 1).

The concordance between ctDNA- and tissue-based NGS results is usually defined as the presence or absence of the identical genomic alteration in a single gene on both molecular platforms. The main causes of discordance between blood and tissue testing are the location and timing of the biopsy, different DNA shedding, tumor heterogeneity and epigenetic modifications. Concordance rates tend to be higher for clonal than subclonal mutations [65,66]. In the situation of ctDNA mutations not found in the matched tumor, subclonal variants may be suspected which were either not present in the collected tissue biopsy, generated by technical errors, or associated with the CHIP. As mentioned before, the inclusion of a white blood cell control sample in the analysis will allow them to be identified. In this study [67], the authors compared 1397 ctDNA samples from metastatic CRC patients (Guardant360, Guardant Health) to three online databases containing tissue-based NGS results (TCGA, NHS/HPFS, GENIE). They only took nonsynonymous, single-nucleotide variants (SNV) into consideration. The result of their analysis showed overall high concordance between ctDNA- and tissue-based NGS results, with a strong correlation between mutation frequency for the top 20 genes in their cohort.

The Korean Lung Liquid versus Invasive Program [68] has recently published a study on 421 samples for which paired ctDNA- and tissue-based NGS results were analyzed. The authors found a rather high concordance (high sensitivity and high specificity of the test) in treatment-naïve patients and could identify additional patients with targetable alterations from the ctDNA results (Guardant360, Guardant Health). However, the specificity rate dropped when tested samples originated from patients who already had EGFRTKI treatment. The fact that clinical responses were observed in patients who carried low-frequency variants (VAF < 1%) which were still detectable by ctDNA analysis underlines the utility of performing such a test routinely. Authors conclude that ctDNA analysis could be used with confidence by clinicians in the case of patients for whom tissue biopsy is not accessible or has already been used in prior testing. Nevertheless, they proposed to prioritize tissue-based testing in these situations where the detection of fusion variants is the primary goal. In the same line, a team from MD Anderson institute [69] analyzed 1971 patients with metastatic NSCLC out of which 217 were paired samples. Their results showed high concordance between ctDNA testing (Guardant360, Guardant Health) and tissue testing (50-gene customized panel or OncoMine Comprehensive Assay V1). 

Another study analyzed the concordance rate in a small cohort of advanced breast cancer patients with either a tissue-based test (Foundation One) or a ctDNA-based test (Guardant360, Guardant Health) [70]. An interesting result presented here was the high concordance when all genes were analyzed, including these with no mutation in either assay. However, the concordance dropped significantly when analyzing a subset of genes with mutations in either assay. This low concordance rate may be explained by the inclusion of not only hotspot mutations but also whole exons and subclones in the analysis. Of note, very high concordance rates observed in other studies usually reflect the nondetection of alterations in either assay. A cohort of 433 patients with different cancer entities was examined and showed similar alteration frequencies for the top 10 genes in both the ctDNA- and tissue-based test [71]. One exception came out with a CDKN2A/B locus which was significantly less observed in ctDNA, probably due to the difficulty of the test to detect allelic loss (Guardant360, Guardant Health) and due to the known challenge of generating good sequence reads for this gene. Further, TP53 alterations’ concordance tended to be higher in metastatic sites compared with the primary tumor, which confirmed the results of another study, in which several alterations presented high concordance rate with metastatic tissue even when primary and metastatic tumors had discordance [72]. In summary, these data show very high overall concordance rates between ctDNA- and tissue-based testing, apart from the detection of EGFR variants acquired during the development of drug resistance (depends on previous therapy lines). However, the positive concordance rates tend to be lower and VAF < 1% still leads to higher discordance, mainly due to tumor heterogeneity. These data also highlight the difficulty to precisely visualize tumor heterogeneity and the need for more studies measuring the impact of this heterogeneity on the clinical outcome.

In a study which compared results of tissue and blood analyses, it has been shown that there is substantial variability in terms of sensitivity (38–89%) and positive predictive values (36–80%) [73]. In addition, most assay discordances appeared at VAF < 1% and originated from technical factors (limited sensitivity, bioinformatic filtering or identification error). These data suggest that the liquid biopsy field would benefit from more rigorous cross-assay comparisons similar to that study.

A common way to circumvent the limitation of the test’s sensitivity is to perform serial ctDNA monitoring or to use prior tumor knowledge in order to increase the confidence of calling low-frequency variants in the cfDNA. This knowledge will help with focusing on known mutated DNA regions, and therefore use smaller NGS panels, and will allow the exclusion of potential CHIP-associated variants [10,74,75,76].

The integration of blood components other than ctDNA in the liquid biopsy tests will increase in the future: miRNAs, circulating tumor cells (CTC), exosomes or tumor-educated platelets, but it should first be validated in clinical trials [77,78,79,80,81]. Techniques other than NGS also present advantages in terms of sensitivity in particular cases (i.e., ddPCR). Recently, an integrative statistical analysis of multiple liquid biopsy analytes was performed on a small, metastatic breast cancer patient cohort [82]. Investigators have shown an additional benefit of combining gDNA, cfDNA, CTC-derived mRNA and extravesicular-derived mRNA.

### 3.3. Confounding Factor: CHIP

The term clonal hematopoiesis of undetermined potential (CHIP) refers to the constant accumulation with age, of mutations in the hematopoietic lineage and in hematopoietic progenitor cells which favors clonal expansion in the blood [83,84,85]. Therefore, these mutations represent a confounding factor when analyzing actual tumor variants without a control sample of white blood cells (WBC). For example, this study used a comprehensive gene panel to identify somatic, nonsynonymous variants in the cfDNA of patients with or without cancer. They found more than 1000 mutations in both groups but also in the additional analysis of the WBC’s DNA. Of note, these mutations included DNMT3A, TET2, PPM1D, and TP53, which are all associated with clonal hematopoiesis [62]. It is therefore highly recommended, especially in the situation of MRD or early cancer detection, to perform additional NGS analysis of white blood cells to be able to exclude CHIP-associated variants.

## 4. Conclusions and Perspectives

In conclusion, detection of ctDNA by NGS has been greatly improved in terms of test performance and allows us to assess the presence of MRD after a curative-intent surgery or treatment. After resolving the above-mentioned limitations, this technique may provide, in the near future, the ability to robustly predict clinical relapses. As a consequence, treatment-associated toxicity will be spared to patients who do not need it (MRD-negative), and adequate adjuvant therapy will be proposed to the others, leading to an increased postoperative overall survival. Current prospectives and randomized clinical trials are assessing whether or not intervention with ctDNA analysis will ultimately improve patient’s outcome by evaluating RFS as a primary endpoint. More interventional studies are required to bring the liquid biopsy field closer to an integration with the clinical routine. Future studies should combine NGS analyses from both tissue and blood, the process of which will need to be tightly set up in terms of timing (rapid enough to inform clinical decisions). Finally, the standardization of pre-analytic processes (sample collection, DNA extraction and quantification) needs to be optimized, and consortia such as the European Liquid Biopsy Society (ELBS) or the International Society of Liquid Biopsy (ISLB) will significantly contribute to improve this situation.

## Figures and Tables

**Table 1:** List of the main NGS-based assays used to assess potential presence of MRD in the blood.

Assay	Company/Reference	Measure	Method	Tumor-Informed Approach
CAPP-Seq	Ref. [12]	large size gene panels	multiplex PCR amplification + ultradeep sequencing	yes
Safe-seq	Ref. [7]	medium size gene panels	deep sequencing with unique molecular identifier (UMI) barcoding	yes
Signatera	Natera	16 somatic variants	multiplex PCR amplification + ultradeep sequencing	yes
PCM	ArcherDX	28 genes	Anchored Multiplex PCR (AMP™) + ultradeep sequencing	yes
RaDaR	Inivata	48 genes	multiplex PCR amplification + ultradeep sequencing	yes
Reveal	Guardant Health	somatic and epigenetic abberations	ultradeep sequencing + bioinformatic classifier to filter non-tumor derived variants	no

**Table 2:** Current clinical trials evaluating the utility of ctDNA detection in the adjuvant setting in NSCLC and CRC. ChT: Chemotherapy, ICI: immune checkpoint inhibitor, AVENIO: Surveillance Kit (ROCHE), LUNAR: Guardant Health, Signatera: Natera.

Entity	Intervention	Platform	Method	ctDNA Marker	Allocation	Acronym	NCT Number
NSCLC	adjuvant ChT or ICI	ArcherDx	PCM	somatic abberations	randomized	MERMAID-I	NCT04385368
NSCLC	adjuvant ChT or ICI	ArcherDx	PCM	somatic abberations	randomized	MERMAID-II	NCT04642469
NSCLC	adjuvant ChT or ICI	AVENIO	NGS	somatic abberations	non-randomized	NA	NCT04585490
NSCLC	adjuvant ICI	AVENIO	NGS	somatic abberations	non-randomized	NA	NCT04585477
NSCLC	adjuvant ChT or ICI	customized	CAPP-Seq	somatic abberations	non-randomized	NA	NCT04367311
NSCLC	observational	customized	CAPP-Seq	somatic abberations	single group	TRACERx	NCT01888601
NSCLC	adjuvant ChT or ICI	Inivata	RaDaR	somatic abberations	randomized	NA	NCT04966663
CRC	adjuvant ChT	LUNAR	NGS	somatic and epigenetic abberations	randomized	COBRA	NCT04068103
CRC	adjuvant ChT	LUNAR	NGS	somatic and epigenetic abberations	NA	PEGASUS	NCT04259944
CRC	adjuvant ChT or ICI	LUNAR	NGS	somatic and epigenetic abberations	randomized	NA	NCT03803553
CRC	observational	customized	NGS	NA	single group	IMPROVE	NCT03637686
CRC	adjuvant ChT	customized	NGS	NA	randomized	IMPROVE-IT	NCT03748680
CRC	adjuvant ChT	NA	Safe-SeqS	somatic abberations	randomized	DYNAMIC-II	ACTRN12615000381583
CRC	adjuvant ChT	NA	Safe-SeqS	somatic abberations	randomized	DYNAMIC-III	ACTRN12617001566325
CRC	adjuvant ChT	Ion Torrent PGM	NGS	somatic abberations	randomized	CIRCULATE	NCT04089631
CRC	adjuvant ChT	Signatera	16-plex PCR/NGS	somatic abberations	randomized	NA	NCT04920032
CRC	personalized cancer vaccine	AVENIO	NGS	somatic abberations	randomized	NA	NCT04486378
CRC	blood multi-analyte collection	LUNAR	NGS	somatic and epigenetic abberations	single group	MiRDA-C	NCT04739072
CRC	observational	Signatera	16-plex PCR/NGS	somatic abberations	single group	TRACC	NCT04050345
CRC	observational	ProBio Trial	NGS	GW CNVs, MSI, and hypermutation	single group	CITCCA	NCT04726800
CRC	observational	Signatera	16-plex PCR/NGS	somatic abberations	single group	BESPOKE	NCT04264702
MSI-H	ICI	F1, G360 or MSK-ACCESS	NGS	somatic abberations	single group	NA	NCT03832569

## Definitions

MRD	minimal residual disease
NGS	next-generation sequencing
ctDNA	circulating tumor DNA
cfDNA	cell-free DNA
gDNA	genomic DNA
CRC	colorectal cancer
NSCLC	non-small-cell lung cancer
FDA	food and drug administration
VAF	variant allele frequency
CAPP-Seq	CAncer Personalized Profiling by deep Sequencing
IDES	integrated digital error suppression
PhasED-Seq	phased variant enrichment and detection sequencing
PPV	positive predictive value
SOC	standard of care
TKI	tyrosine kinase inhibitor
ICI	immune checkpoint inhibitor
PFS	progression free survival
OS	overall survival
TMB	tumor mutational burden
MSI	microsatellite instability
CRT	chemoradiotherapy
DCB	durable clinical benefit
CHIP	clonal hematopoiesis of undetermined potential
WBC	white blood cells
HRD	homologous recombination deficiency
CTC	circulating tumor cells
RFS	relapse-free survival
